# Additions to the Genus *Arthrinium* (Apiosporaceae) From Bamboos in China

**DOI:** 10.3389/fmicb.2021.661281

**Published:** 2021-04-07

**Authors:** Yao Feng, Jian-Kui (Jack) Liu, Chuan-Gen Lin, Ya-Ya Chen, Mei-Mei Xiang, Zuo-Yi Liu

**Affiliations:** ^1^College of Agriculture, Guizhou University, Guiyang, China; ^2^Guizhou Key Laboratory of Agricultural Biotechnology, Guizhou Academy of Agricultural Sciences, Guiyang, China; ^3^School of Life Sciences and Technology, University of Electronic Science and Technology, Chengdu, China; ^4^Centre of Excellence in Fungal Research, Mae Fah Luang University, Chiang Rai, Thailand; ^5^Institute of Crop Germplasm Resources, Guizhou Academy of Agricultural Sciences, Guiyang, China; ^6^Innovative Institute for Plant Health, Zhongkai University of Agriculture and Engineering, Guangzhou, China

**Keywords:** 4 new taxa, asexual-sexual morphs, multi-genes, phylogeny, taxonomy

## Abstract

*Arthrinium* has a widespread distribution occurring in various substrates (e.g., air, soil debris, plants, lichens, marine algae and even human tissues). It is characterized by the basauxic conidiogenesis in the asexual morph, with apiospores in the sexual morph. In this study, seventeen isolates of *Arthrinium* were collected in China. Based on their morphology and phylogenetic characterization, four new species (*A. biseriale*, *A. cyclobalanopsidis*, *A. gelatinosum*, and *A. septatum*) are described and seven known species (*A. arundinis*, *A. garethjonesii*, *A. guizhouense*, *A. hydei*, *A. neosubglobosa*, *A. phyllostachium* and *A. psedoparenchymaticum*) are identified, of which the sexual morph of three species (*A. guizhouense*, *A. phyllostachium* and *A. psedoparenchymaticum*) and asexual morph of *A. garethjonesii* are reported for the first time. The detailed descriptions, illustrations and comparisons with related taxa of these new collections are provided. Phylogenetic analyses of combined ITS, LSU, TUB2, and TEF sequence data support their placements in the genus *Arthrinium* and justify the new species establishments and identifications of known species.

## Introduction

The genus *Arthrinium* Kunze belongs to the family Apiosporaceae, which was introduced by Hyde et al. (1998) and typified by the genus *Apiospora* (Ellis, 1971; Seifert et al., 2011; Hyde et al., 2020). *Arthrinium* is the largest genus within Apiosporaceae and it has a widespread distribution on variety of hosts, 77 species have been recorded by Species Fungrom (March, 2021). Classification of *Arthrinium* was primarily based on conidial shape, conidiophores, sterile cells, and the presence of setae. Some morphologically different taxa (genera) grouped with *Arthrinium* when use the molecular data (Crous and Groenewald, 2013). Thus, those characteristics could be not fully inferred about the phylogenetic relationships for *Arthrinium* (Crous and Groenewald, 2013; Wang et al., 2018; Pintos et al., 2019). Except for being reported as saprobes (Agut and Calvo, 2004; Crous and Groenewald, 2013; Dai et al., 2016, 2017; Jiang et al., 2018; Wang et al., 2018; Jiang et al., 2019, 2020; Luo et al., 2019; Pintos et al., 2019; Yan et al., 2019; Mapook et al., 2020; Senanayake et al., 2020a; Tang et al., 2020), the species of *Arthrinium* also includes phytopathogenic fungi, for instance, *A. arundinis* causing brown culm streak of *Phyllostachys praecox, A. phaeospermum* causing culm rot on *Phyllostachys viridis* and cutaneous infections of humans (Rai, 1989; Zhao et al., 1990; Martínez-Cano et al., 1992; Mavragani et al., 2007; Crous and Groenewald, 2013; Li et al., 2016; Wang et al., 2018).

we are carrying out the survey of fungal diversity in Karst formations of the Asian region, and many new taxa are described in last few years (Li et al., 2016; Chen et al., 2017, 2020; Zhang et al., 2017, 2019; Feng et al., 2019; Liu et al., 2019; Dissanayake et al., 2020b). In this study, seventeen *Arthrinium*-like were collected in Guizhou and Guangdong province, China and can be recognized as eleven *Arthrinium* species based on morphological characters and phylogeny inferred from the multi-gene sequences data (ITS, LSU, TUB2, and TEF) analyses, which four new species (*A. biseriale*, *A. cyclobalanopsidis*, *A. gelatinosum*, and *A. septatum*) and seven known species (*A. arundinis*, *A. garethjonesii*, *A. guizhouense*, *A. hydei*, *A. neosubglobosa*, *A. phyllostachium* and *A. psedoparenchymaticum*) are introduced and identified, respectively. The aim of this study is to describe these new taxa with detailed descriptions and illustrations, and also provide their phylogenetic relationships within *Arthrinium* based on multi-gene analysis.

## Materials and Methods

### Sample Collection, Morphological Studies and Isolation

Samples were collected from Guizhou and Guangdong Province in China. Fungal fruiting bodies were examined by using stereomicroscope (Motic SMZ 168). Free hand sections of fungal structures were mounted in water for microscopic studies and photomicrography. Images were taken by using a Nikon ECLIPSE Ni compound microscope fitted with a Canon EOS 70D digital camera. All measurements were taken by using Tarosoft Image Frame Work software (IFW) (Liu et al., 2010), and photo plates were processed with Adobe Photoshop CS6 software (Adobe Systems, United States).

The single spore isolation followed the method described in Senanayake et al. (2020b). Parts of morphological descriptions were based on sporulated cultures on WA (Water Agar) at room temperature (ca. 25°C). Type specimens were deposited in the herbarium of Cryptogams Kunming Institute of Botany Academia Sinica (HKAS), Kunming, China and Guizhou Academy of Agriculture sciences Herbarium (GZAAS). Pure cultures were deposited in China General Microbiological Culture Collection Center (CGMCC) and Guizhou Culture Collection (GZCC). Faces of Fungi^1^ number is obtained as described in the paper by Jayasiri et al. (2015), and the new taxa are registered in Index Fungorum (2021).

### DNA Extraction, PCR Amplification and Sequencing

Fungal mycelia were scraped from the pure culture which were growing on PDA (Potato Dextrose Agar) for one week at 25°C in dark. DNA was extracted by using Ezup Column Fungi Genomic DNA Purification Kit (Sangon Biotech, China) from fresh fungal mycelia, but some were extracted directly from fruiting bodies by using Forensic DNA Kit (Omega Bio-Tek, China). Four gene regions, large subunit rDNA (LSU), internal transcribed spacer (ITS), beta-tubulin (TUB2) and the translation elongation factor 1-alpha (TEF) gene were amplified by the primer pairs LR0R and LR5 (Vilgalys and Hester, 1990), ITS5 and ITS4, T1 (O’Donnell and Cigelnik, 1997) and Bt2b (Glass and Donaldson, 1995), EF1-728F and EF-2 (O’Donnell et al., 1998; Carbone and Kohn, 1999), respectively. Polymerase chain reaction (PCR) was carried out in 25 μL reaction volume containing 12.5 μL 2 × PCR Master Mix (Sangon Biotech, China), 9.5 μL ddH_2_O, 1 μL of each primer and 1 μL DNA template. The annealing temperatures were adjusted to 56°C for ITS, LSU and TUB2, and 55°C for TEF. PCR products were sent to sequence at Sangon Biotech Co., Ltd., China. The PCR products were examined using 1.2% agarose electrophoresis gel stained with ethidium bromide. PCR products were purified and sequenced by Sangon Biotech (Shanghai) Co., Ltd., China. New generated nucleotide sequences were submitted in GenBank (Table 1).

**TABLE 1:** GenBank accession numbers of species included in the phylogenetic study.

Species	Strain no.	GenBank Accession Numbers			
		LSU	ITS	TEF	TUB2
*Arthrinium acutiapicum*	KUMCC 20–0209	MT946338	MT946342	MT947359	MT947365
*A. acutiapicum*	KUMCC 20–0210	MT946339	MT946343	MT947360	MT947366
*A. aquaticum*	MLFU 18–1628	MK835806	MK828608	N/A	N/A
*A. arundinis*	CBS 106.12	KF144927	KF144883	KF145015	KF144973
*A. arundinis*	CBS 114316	KF144928	KF144884	KF145016	KF144974
***A. arundinis***	**GZCC 20–0116**	**MW478899**	**MW481720**	**MW522952**	**MW522968**
*A. aureum*	CBS 244.83	KF144935	AB220251	KF145023	KF144981
*A. balearicum*	CBS 145129	MK014836	MK014869	MK017946	MK017975
*A. bambusae*	LC7107	N/A	KY494719	KY705117	KY705187
*A. bambusae*	LC7106	KY494794	KY494718	KY806204	KY705186
*A. bambusicola*	MFLUCC 20–0144	MW173087	MW173030	MW183262	N/A
***A. biseriale***	**CGMCC 3.20135**	**MW478885**	**MW481708**	**MW522938**	**MW522955**
***A. biseriale***	**GZCC 20–0099**	**MW478886**	**MW481709**	**MW522939**	**MW522956**
***A. biseriale***	**GZCC 20–0100**	**MW478887**	**MW481710**	**MW522940**	**MW522957**
*A. camellia-sinensis*	LC8181	KY494837	KY494761	KY705157	KY705229
*A. camellia-sinensis*	LC5007	KY494780	KY494704	KY705103	KY705173
*A. caricicola*	CBS 145127	MK014838	MK014871	MK017948	MK017977
*A. chinense*	CFCC53036	N/A	MK819291	MK818545	MK818547
*A. chinense*	CFCC53037	N/A	MK819292	MK818546	MK818548
*A. chromolaenae*	MFLUCC 17-1505	MT214436	MT214342	N/A	N/A
***A. cyclobalanopsidis***	**CGMCC 3.20136**	**MW478892**	**MW481713**	**MW522945**	**MW522962**
***A. cyclobalanopsidis***	**GZCC 20–0103**	**MW478893**	**MW481714**	**MW522946**	**MW522963**
*A. descalsii*	CBS 145130	MK014837	MK014870	MK017947	MK017976
*A. dichotomanthi*	LC8175	KY494831	KY494755	KY705151	kY705223
*A. dichotomanthi*	LC4950	KY494773	KY494697	KY705096	KY705167
*A. esporlense*	CBS 145136	MK014845	MK014878	MK017954	MK017983
*A. euphorbiae*	IMI 285638b	AB220335	AB220241	N/A	AB220288
*A. gaoyouense*	CFCC 52301	N/A	MH197124	MH236793	MH236789
*A. gaoyouense*	CFCC 52302	N/A	MH197125	MH236794	MH236790
*A. garethjonesii*	KUMCC16–0202	KY356091	KY356086	N/A	N/A
***A. garethjonesii***	**GZCC 20–0115**	**MW478894**	**MW481715**	**MW522947**	**N/A**
***A. gelatinosum***	**KHAS 11962**	**MW478888**	**MW481706**	**MW522941**	**MW522958**
***A. gelatinosum***	**GZAAS 20–0107**	**MW478889**	**MW481707**	**MW522942**	**MW522959**
*A. guizhouense*	LC5318	KY494784	KY494708	KY705107	KY705177
*A. guizhouense*	LC5322	KY494785	KY494709	KY705108	KY705178
***A. guizhouense***	**GZCC 20–0114**	**MW478895**	**MW481716**	**MW522948**	**MW522964**
*A. gutiae*	CBS 135835	KR149063	KR011352	KR011351	KR011350
*A. hispanicum*	IMI 326877	AB220336	AB220242	N/A	AB220289
*A. hydei*	CBS 114990	KF144936	KF144890	KF145024	KF144982
*A. hydei*	KUMCC 16–0204	KY356092	KY356087	N/A	N/A
***A. hydei***	**GZCC 20–0113**	**MW478900**	**MW481721**	**MW522953**	**N/A**
*A. hyphopodii*	KUMCC 16–0201	KY356093	KY356088	N/A	N/A
*A. hyphopodii*	MFLUCC15–0003	N/A	KR069110	N/A	N/A
*A. hysterinum*	CBS 145134	MK014843	MK014876	N/A	N/A
*A. hysterinum*	ICMP 6889	MK014841	MK014874	MK017951	MK017980
*A. ibericum*	CBS 145137	MK014846	MK014879	MK017955	MK017984
*A. italicum*	CBS 145138	MK014847	MK014880	MK017956	MK017985
*A. japonicum*	IFO 30500	AB220356	AB220262	N/A	AB220309
*A. japonicum*	IFO 31098	AB220358	AB220264	N/A	AB220311
*A. jatrophae*	MMI 00052	N/A	JQ246355	N/A	N/A
*A. jatrophae*	AMH–9557	N/A	JQ246355	N/A	N/A
*A. jiangxiense*	LC4577	KY494769	KY494693	KY705092	KY705163
*A. jiangxiense*	LC4578	KY494770	KY494694	KY705093	KY705164
*A. kogelbergense*	CBS 113332	KF144937	KF144891	KF145025	KF144983
*A. kogelbergense*	CBS 113333	KF144938	KF144892	KF145026	KF144984
*A. locuta-pollinis*	LC11683	N/A	MF939595	MF939616	MF939622
*A. longistromum*	MFLUCC 11–0481	KU863129	KU940141	N/A	N/A
*A. longistromum*	MFLUCC 11–0479	KU863130	KU940142	N/A	N/A
*A. malaysianum*	CBS 102053	KF144942	KF144896	KF145030	KF144988
*A. malaysianum*	CBS 251.29	KF144943	KF144897	KF145031	KF144989
*A. marii*	CBS 497.90	KF144947	AB220252	KF145035	KF144993
*A. mediterranei*	IMI 326875	AB220337	AB220243	N/A	AB220290
*A. minus*	CBS 145131	MK014839	MK014872	MK017949	MK017978
*A. mytilomorphum*	DAOM 214595	N/A	KY494685	N/A	N/A
*A. garethjonesii*	KUMCC 18–0192	MK070898	MK070897	N/A	N/A
*A. neosubglobosa*	KUMCC 16–0203	KY356095	KY356090	N/A	N/A
*A. neosubglobosa*	JHB006	KY356094	KY356089	N/A	N/A
***A. neosubglobosa***	**GZAAS 20–0099**	**MW478901**	**MW481705**	**MW522954**	**MW522969**
*A. obovatum*	LC8177	KY494833	KY494757	KY705153	KY705225
*A. obovatum*	LC4940	KY494772	KY494696	KY705095	KY705166
*A. ovatum*	CBS 115042	KF144950	KF144903	KF145037	KF144995
*A. paraphaeospermum*	MFLUCC 13–0644	KX822124	KX822128	N/A	N/A
*A. phaeospermum*	CBS 114314	KF144951	KF144904	KF145038	KF144996
*A. phaeospermum*	CBS 114315	KF144952	KF144905	KF145039	KF144997
*A. phragmites*	CPC 18900	KF144956	KF144909	KF145043	KF145001
*A. phyllostachium*	MFLUCC 18–1101	MH368077	MK351842	MK340918	MK291949
***A. phyllostachium***	**GZCC 20–0111**	**MW478896**	**MW481717**	**MW522949**	**MW522965**
***A. phyllostachium***	**GZCC 20–0112**	**MW478897**	**MW481718**	**MW522950**	**MW522966**
*A. piptatheri*	CBS 145149	MK014860	MK014893	MK017969	N/A
*A. pseudoparenchymaticum*	LC7234	KY494819	KY494743	KY705139	KY705211
*A. pseudoparenchymaticum*	LC8173	KY494829	KY494753	KY705149	KY705221
***A. pseudoparenchymaticum***	**GZCC 20–0117**	**MW478898**	**MW481719**	**MW522951**	**MW522967**
*A. pseudorasikravindrae*	KUMCC 20–0208	N/A	MT946344	MT947361	MT947367
*A. pseudorasikravindrae*	KUMCC 20–0211	N/A	MT946345	MT947362	MT947368
*A. pseudosinense*	CPC 21546	KF144957	KF144910	KF145044	N/A
*A. pseudosinense*	CBS 135459	N/A	KF144910	KF145044	N/A
*A. pseudospegazzinii*	CBS 102052	KF144958	KF144911	KF145045	KF145002
*A. pterospermum*	CBS 123185	KF144959	KF144912	N/A	KF145003
*A. pterospermum*	CPC 20193	KF144960	KF144913	KF145046	KF145004
*A. puccinioides*	CBS 145150	MK014861	MK014894	MK017970	MK017998
*A. puccinioides*	CBS 549.86	AB220347	AB220253	N/A	AB220300
*A. qinlingense*	CFCC 52303	N/A	MH197120	MH236795	MH236791
*A. qinlingense*	CFCC 52304	N/A	MH197121	MH236796	MH236792
*A. rasikravindrae*	CBS 145152	MK014863	MK014896	MK017971	MK017999
*A. rasikravindrae*	LC7115	KY494797	KY494721	KY705118	KY705189
*A. rasikravindrae*	NFCCI 2144	N/A	JF326454	N/A	N/A
*A. sacchari*	CBS 212.30	KF144963	KF144917	KF145048	KF145006
*A. sacchari*	CBS 301.49	KF144962	KF144916	KF145047	KF145005
*A. saccharicola*	CBS 191.73	KF144966	KF144920	KF145051	KF145009
*A. saccharicola*	CBS 463.83	KF144968	KF144921	KF145053	KF145011
***A. septatum***	**CGMCC 3.20134**	**MW478890**	**MW481711**	**MW522943**	**MW522960**
***A. septatum***	**GZCC 20–0109**	**MW478891**	**MW481712**	**MW522944**	**MW522961**
*A. serenense*	IMI 326869	AB220344	AB220250	N/A	AB220297
*A. setostromum*	KUMCC 19–0217	MN528011	MN528012	MN527357	N/A
*A. sporophleum*	CBS 145154	MK014865	MK014898	MK017973	MK018001
*A. subglobosum*	MFLUCC 11–0397	KR069113	KR069112	N/A	N/A
*A. subroseum*	LC7292	KY494828	KY494752	KY705148	KY705220
*A. thailandicum*	MFLUCC 15–0199	KX986111	KU940146	N/A	N/A
*A. thailandicum*	MFLUCC 15–0202	KU863133	KU940145	N/A	N/A
*A. trachycarpum*	CFCC 53038	N/A	MK301098	MK303396	MK303394
*A. trachycarpum*	CFCC 53039	N/A	MK301099	MK303397	MK303395
*A. urticae*	IMI 326344	AB220339	AB220245	N/A	N/A
*A. vietnamese*	IMI 99670	KX986111	KX986096	N/A	KY019466
*A. xenocordella*	CBS478.86	KF144970	KF144925	KF145055	KF145013
*A. xenocordella*	CBS 595.66	KF144971	KF144926	N/A	N/A
*A. yunnanum*	DDQ 00281	KU863136	KU940148	N/A	N/A
*A. yunnanum*	MFLU 15–0002	N/A	KU940147	N/A	N/A
*Seiridium phylicae*	CPC 19962	NG 042759	LT853092	LT853189	LT853239
*Seiridium phylicae*	CPC 19965	KC005809	LT853093	LT853190	LT853240

*The newly generated sequence is shown in bold. AMH: Ajrekar Mycological herbarium, Pune, Maharashtra, India; CBS: Westerdijk Fungal Biodiversity Institute, Utrecht, Netherlands; CFCC: China Forestry Culture Collection Center, Beijing, China; CGMCC: China General Microbiological Culture Collection Center, Beijing, China; CPC: Culture collection of Pedro Crous, housed at the Westerdijk Fungal Biodiversity Institute; DAOM: Canadian Collection of Fungal Cultures, Ottawa, Canada; DDQ: D.Q. Dai; GZAAS: Guizhou Academy of Agricultural Sciences herbarium, China; GZCC: Guizhou Culture Collection, China; HKAS: Herbarium of Cryptogams Kunming Institute of Botany Academia Sinica; ICMP: International Collection of Microorganisms from Plants, New Zealand; IFO: Institute for Fermentation, Osaka, Japan; IMI: Culture collection of CABI Europe UK Centre, Egham, UK; JHB: H.B. Jiang; KUMCC: Culture collection of Kunming Institute of Botany, Yunnan, China; LC: Working collection of Lei Cai, housed at CAS, China; MFLUCC: Mae Fah Luang University Culture Collection, Chiang Rai, Thailand; NFCCI: National Fungal Culture Collection of India.*

### Phylogenetic Analyses

Phylogenetic analyses were performed based on ITS, LSU, TUB2 and TEF sequence data. The related strains of Apiosporaceae (Table 1) used for analysis were referred to BLAST^2^ results and relevant publications (Wang et al., 2018; Jiang et al., 2019, 2020; Pintos et al., 2019; Tang et al., 2020). Sequences were obtained from GenBank and aligned using MAFFT v. 7 (Katoh and Standley, 2013). Manual adjustment was also performed when it is necessary by using BioEdit v. 7.0 (Hall, 1999). The alignment of sequences data used in analyses is deposited in TreeBASE under the accession number S27728. The analyses of maximum parsimony (MP), maximum likelihood (ML) and Bayesian inference (BI) were carried out as detailed in Dissanayake et al. (2020a) and programs used including PAUP v.4.0b 10 (Hillis and Bull, 1993; Swofford, 2002), raxmlGUI v. 1.3 (Silvestro and Michalak, 2012), and MrBayes v3.1.2 (Rannala and Yang, 1996; Huelsenbeck and Ronquist, 2001; Zhaxybayeva and Gogarten, 2002; Nylander, 2004). Trees were visualized with FigTree v1.4.2 (Rambaut, 2012) and the layout was edited using Adobe Illustrator CS6.

## Results

### Phylogeny

**FIGURE 1:**
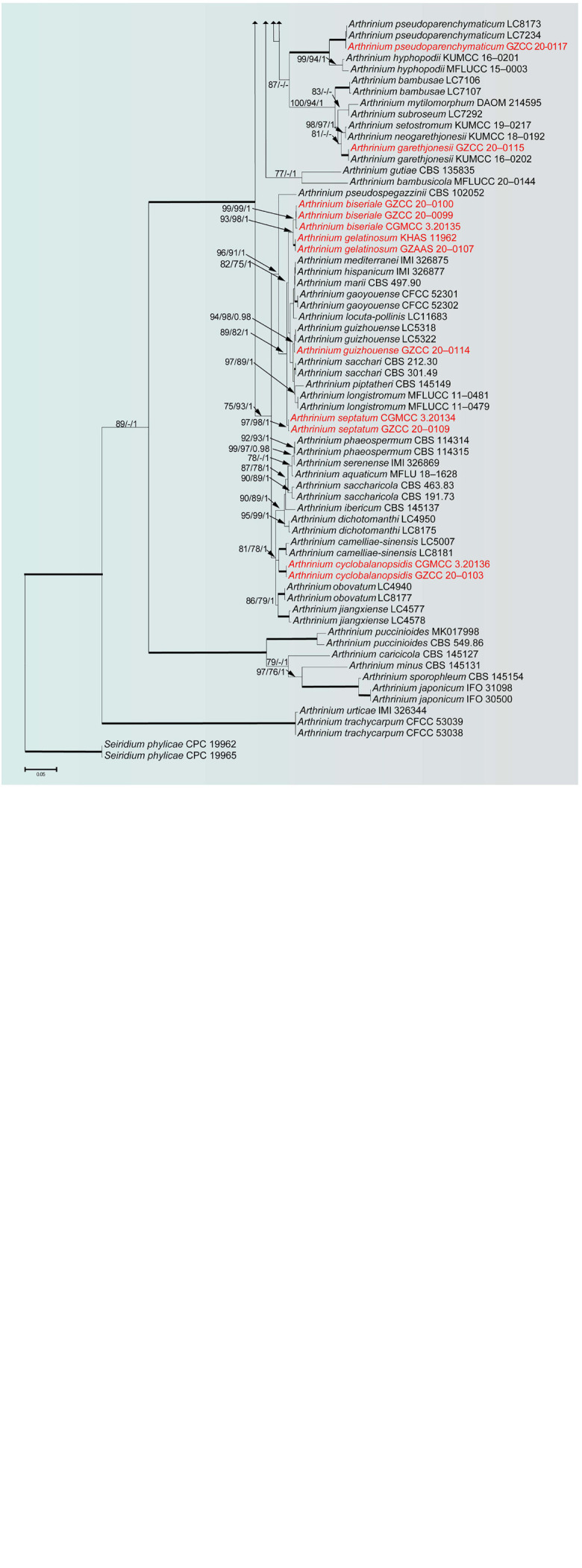
RAxML tree of representatives based on a combined dataset of ITS, LSU, TUB2 and TEF sequences. Bootstrap support values for ML, MP (≥75%) and Bayesian (≥0.95) are given at the nodes (ML/MP/BI). Branches with ML, MP and BI equal 100, 100 and 1 are in bold. The tree is rooted with *Seiridium phylicae* (CPC 19962 and CPC 19965). New strains are shown in red.

To determine the phylogenetic placement of the new collections in this study, the combined ITS, LSU, TUB2 and TEF data set comprised 119 taxa with *Seiridium phylicae* (CPC 19962 and CPC 19965) as the outgroup taxa. The concatenated alignment comprises 2,770 characters (ITS: 1–635; LSU: 636–1,454; TUB2: 1,455–2,300; TEF: 2,301–2,770) including gaps, of which 1,361 characters were constant, and 1,279 characters are parsimony informative and 130 are parsimony uninformative. Maximum likelihood, maximum parsimony and Bayesian analyses were performed, respectively, and presented consistent topologies. The best scoring RAxML tree (Figure 1) is obtained with a final likelihood value of -27544.434044. Estimated base frequencies were as follows: A = 0.236829, C = 0.253015, G = 0.251523, T = 0.258634; substitution rates AC = 1.154254, AG = 2.738844, AT = 1.073582, CG = 0.896658, CT = 4.134885, GT = 1.000000; The gamma distribution shape parameter alpha is equal to 0.332641 and the Tree-Length equal to 3.795226.

Phylogenetic analyses showed that our newly collected seventeen taxa clustered into eleven clades and can be recognized as seven known species (*Arthrinium arundinis*, *A. garethjonesii*, *A. guizhouense*, *A. hydei*, *A. neosubglobosa*, *A. phyllostachium*, *A. psedoparenchymaticum*) and four new species (*A. biseriale*, *A. cyclobalanopsidis*, *A. gelatinosum*, *A. septatum*) (Figure 1).

### Taxonomy

#### *Arthrinium biseriale* Y. Feng and Z.Y. Liu, sp. nov. Figure 2

Index Fungorum number: IF558136

**FIGURE 2:**
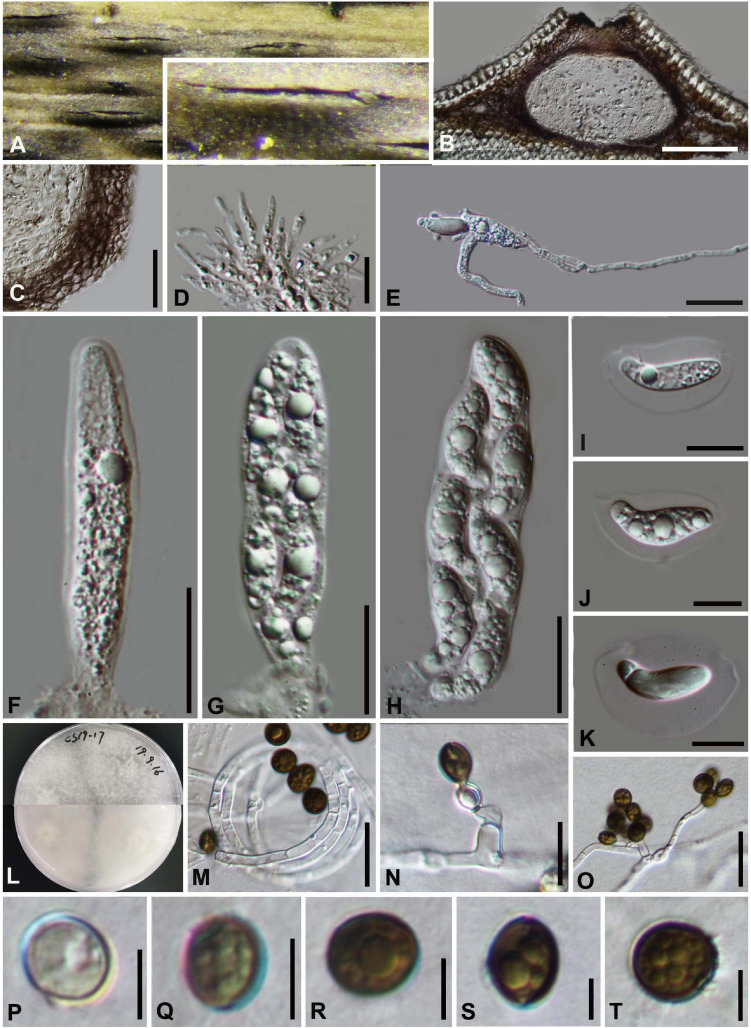
***Arthrinium biseriale*** (HKAS 111961**, holotype**). **(A)** Appearance of stromata on bamboo host. **(B)** Vertical section of stroma. **(C)** Peridium. **(D)** Paraphyses. **(E)** Germinating ascospore. **(F–H)** Asci. **(I–K)** Ascospore. **(L)** Culture. **(M)** Hyphae. (**N**,**O)** Conidiophore and conidiogenous cells. (**P–T)** Conidia. **Scale bars: (B)** = 50 μm. **(C)** = 20 μm. **(D)** = 10μm. **(E–H)** = 20 μm. **(I–K)** = 10 μm. **(M)** = 20 μm. **(N)** = 10 μm. **(O)** = 20 μm. **(P–T)** = 5 μm.

Facesoffungi number: FoF 09569

Etymology: The epithet refers to the ascospores are arranged in two rows in the ascus.

Holotype: HKAS 111961

*Saprobic* on dead bamboo culms, forming black, lenticular spots on the host surface, with stromata breaking through raised cracks with black center, and merge with each other with age, forming an erumpent black mass visible at the naked eye. **Sexual morph:** *Stromata* scattered to gregarious, immersed to erumpent, later becoming superficial, dark brown to black, fusiform, forming a slit-like opening at the apex, multi-loculate, membranous, with a periphysate ostiole. *Ascomata* 122–153 μm high, 138–207 μm diam, arranged in rows, dark brown to black. *Peridium* 11–19 μm wide, composed of several layers of dark brown to hyaline cells of *textura angularis*. *Hamathecium* 3–4 μm wide, comprising dense, hyaline, septa paraphyses. *Asci* 84–116 μm × 18–25 μm ($\bar{x}$ = 97 μm × 21 μm, *n* = 20), 8-spored, unitunicate, clavate, apically rounded, with an indistinct pedicel. *Ascospores* 22–28 μm × 7–11 μm ($\bar{x}$ = 25 μm × 9 μm, *n* = 30), biseriate, fusiform, curved at the bottom, obtuse at both ends, slightly wider in the middle, hyaline, 1-septate, constricted at the septum, mostly curved at the lower cell, rarely straight, with a large upper cell and a small lower cell, smoothwalled. The lower cell has 1-guttulate, the upper cell has 1–3 big guttulate in the middle surrounded by multiple small guttulate with a shallow 4–7 μm thick gelatinous sheath in the early. Growing to a later stage, the guttulate filled the entire spore, and the gelatinous sheath dissolves easily. **Asexual morph:** On WA, *Hyphae* 2.5–6.0 μm diam, hyaline, branched, septate, some curled in a ring structure. *Conidiophores* 12.0–44.0 μm × 2.5–5.0 μm ($\bar{x}$ = 20.0 μm × 3.5 μm, *n* = 20), straight or flexuous, smooth, thin-walled, unbranched, hyaline to pale brown, cylindrical, cyathiform, having transverse septa, often reduced to conidiogenous cells. *Conidiogenous cells* 5.0–22.0 μm × 2.5–5.0 μm ($\bar{x}$ = 10.0 μm × 3.5 μm, *n* = 20), integrated, hyaline to pale brown, doliiform to ampulliform, or lageniform. *Conidia* 7–9 μm long (*n* = 30), brown, smooth in surface view, and 7–11 μm long (*n* = 30), lenticular, with a paler equatorial slit in side view, globose to ellipsoid with many guttules.

##### Culture characters

Ascospores germinated on WA within 24 h and germ tubes produced from middle and lower end. Colonies fast grown on PDA at 25°C, reached 7 cm in 7 days at 25°C. Colonies evenly tiled, with a large number of aerial hyphae, white, velvety, thin, gray-white on the reverse side and dirty white in the center.

##### Materials examined

**China**, Guizhou Province, Chishui City, Zhuhai National Forest Park, on dead culms of bamboo, 10 July 2019, Yao Feng, CS19-17 (HKAS 111961, holotype; GZAAS 20–0102, isotype), ex-type living cultures, CGMCC 3.20135 = GZCC 20–0101. *Ibid*., on dead base of the bamboo stem, 10 July 2019, Yao Feng, CS 19-25 (GZAAS 20–0101), living culture, GZCC 20–0100. *Ibid*., on dead branch of bamboo, 11 July 2019, Ya-Ya Chen, CS 013 (GZAAS 20–0100), living culture, GZCC 20–099.

##### Notes

Three strains representing *Arthrinium biseriale* clustered in a well-supported clade which are closely related to *A. gelatinosum*, but phylogenetically distinct and can be recognized as two different species (99% sequence similarity in ITS; 99% in TEF; 98% in TUB2). Morphologically, *Arthrinium biseriale* has smaller stromata (122–153 μm × 138–207 μm vs. 144–199 μm × 184–214 μm) and the spores of *A. biseriale* are more curved than those of *A. gelatinosum*.

#### *Arthrinium gelatinosum* Y. Feng and Z.Y. Liu, sp. nov. Figure 3

Index Fungorum number: IF558137

**FIGURE 3:**
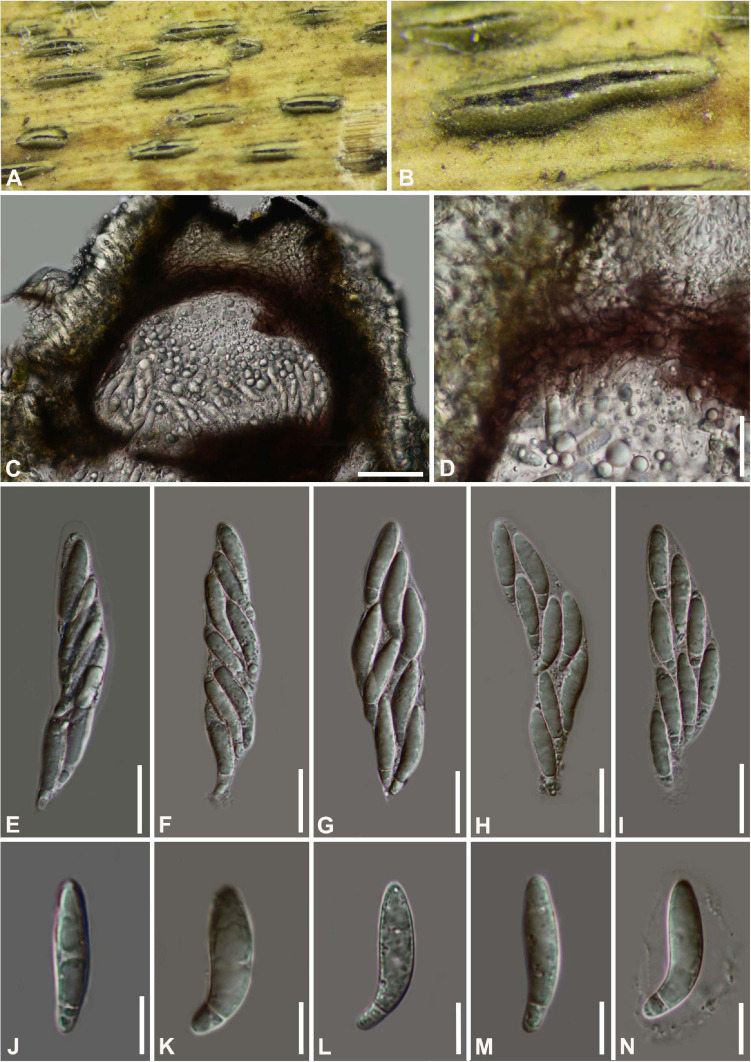
***Arthrinium gelatinosum*** (HKAS 111962, **holotype)**. **(A,B)** Appearance of stromata on bamboo host. **(C)** Vertical section of stroma. **(D)** Peridium. **(E–I)** Asci. **(J–N)** Ascospore. **Scale bars: (C)** = 50 μm. **(D–I)** = 20 μm. **(J–N)** = 10 μm.

Facesoffungi number: FoF 09570

Etymology: The epithet refers to the ascospore surrounded by gelatinous sheath.

Holotype: HKAS 111962

*Saprobic* on dead bamboo culms, forming black, lenticular spots on the host surface, with stromata breaking through raised cracks with black center. **Sexual morph:** *Stromata* solitary to gregarious, immersed to erumpent, fusiform, with long axis broken at the top by one cracks. *Ascomata* 144–199 μm high μm × 184–214 μm wide, uniseriate or irregularly arranged beneath stromata, pseudothecial, black, globose to subglobose with a flattened base. *Peridium* composed of 5 or 6 layers of brown cells arranged in *textura angularis*, with a conspicuous perfusate ostiole. *Hamathecium paraphyses* hyphae-like. *Asci* 85–121 μm × 15–24 μm ($\bar{x}$ = 100 μm × 17 μm, *n* = 20), 8-spored, unitunicate, clavate, apically rounded, broadly cylindrical, with an indistinct pedicel. *Ascospores* (27−) 28-31 (−32) μm × 6–8 μm ($\bar{x}$ = 30 μm × 7 μm, *n* = 30), apiosporic, clavate to fusiform with narrowly rounded ends, composed of a large guttate and small guttate, hyaline, smooth-walled, surrounded by a gelatinous sheath. Growing to a later stage, the guttate filled the entire spore, and the gelatinous sheath dissolves easily. **Asexual morph:** undetermined.

##### Materials examined

**China**, Guizhou Province, Chishui City, Zhuhai National Forest Park, on dead culms of bamboo, 10 July 2019, Yao Feng, CS19-32 (HKAS 111962, holotype; GZAAS20–0108, isotype). *Ibid*., Chishui National Scenic Area, on dead branch of bamboo, 10 July 2019, Yao Feng, CS 19–29 (GZAAS 20–0107).

##### Notes

Two taxa representing *Arthrinium gelatinosum* cluster in a well-supported lineage (ML/MP/BI = 93/98/1, Figure 1), which is a sister to *A. biseriale*, and they are phylogenetically distinct species.

#### *Arthrinium septatum* Y. Feng and Jian K. Liu, sp. nov. Figure 4

Index Fungorum number: IF558138

**FIGURE 4:**
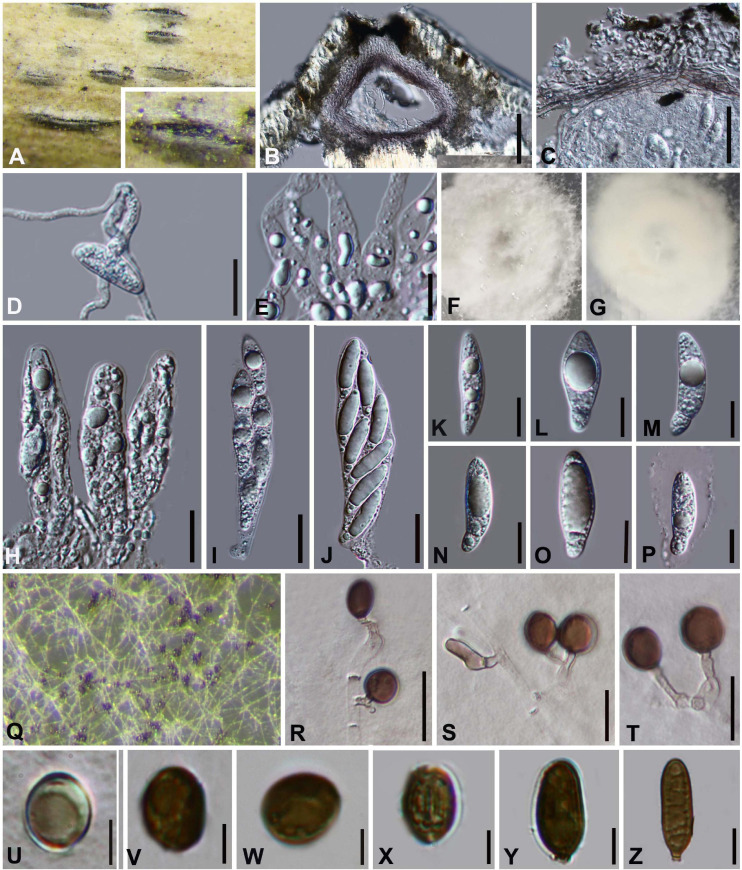
***Arthrinium septatum*** (HKAS 111960, **holotype)**. **(A)** Appearance of stromata on bamboo host. **(B)** Vertical section of stroma. **(C)** Peridium. **(D)** Germinating ascospore. **(E)** Paraphyses. (**F**,**G)** Culture. **(H–J)** Asci. **(K–P)** Ascospore. **(Q)** Colony on PDA producing conidia masses. **(R–T)** Conidiophore and conidiogenous cells. **(U–X)** Conidia. **(Y,Z)** Sterile cell. **Scale bars: (B)** = 50 μm. **(C,D)** = 25 μm. **(E)** = 10 μm. **(H–J)** = 20 μm. **(K–P)** = 10 μm. **(R–T)** = 10 μm. **(U–Z)** = 5 μm.

Facesoffungi number: FoF 09571

Etymology: The epithet refers to the septate conidiophore.

Holotype: HKAS 111960

*Saprobic* on dead bamboo culms. **Sexual morph:** *Stromata* scattered to gregarious, immersed to erumpent, initially breaks through a black spot on the host, later visible as black, raised, lenticular or dome-shaped, and will grow into a linear shape at the later stage of growth. Ostiolate, with the long axis broken at the top revealing the ostioles of pseudothecia. *Ascomata* 88–195 μm high × 160–185 μm wide ($\bar{x}$ = 140 μm × 173 μm, *n* = 10), arranged in rows, brown to dark brown, subglobose with a flattened base. *Peridium* with several layers of cells arranged in *textura angularis*, with a conspicuous ostiole 50–90 μm in diameter, periphysate. *Hamathecium paraphyses* hyphae-like, septate, hyaline. *Asci* 75–104 μm × 17–26 μm ($\bar{x}$ = 91 μm × 20 μm, *n* = 20), 8-spored, clavate, cylindrical, apically rounded, with distinct pedicel. *Ascospores* (24−) 25–30 (−32) μm × (6−) 8–10 (−11) μm ($\bar{x}$ = 29 μm × 9 μm, *n* = 30), biseriate, broad fusiform to cylindrical, with a large upper cell and a small lower cell, hyaline, 1-septate, constricted at septum, slightly curved, smooth-walled, with many guttules, with a large guttule at the center of large upper cell, with a distinct gelatinous sheath. **Asexual morph:** On PDA, *Hyphae* 2–4 μm in diameter, hyaline, branched, septate. *Conidiophores* 12.0–63.0 × 2–5 ($\bar{x}$ = 313 μm × 3 μm, *n* = 20), straight or flexuous, smooth, thin-walled, septate, hyaline to light brown, cylindrical, sometimes reduced to conidiogenous cells. *Conidiogenous cells* 4.0–18.0 × 1.5–4.0 ($\bar{x}$ = 10.0 μm × 2.5 μm, *n* = 20), solitary on hyphae, integrated, branched, ampuliform, cylindrical, hyaline to brown. *Conidia* 8–11 (−13) μm long (*n* = 30), brown, smooth, guttulate, globose to ellipsoid in surface view. and (8−) 9–13 (−14) μm long (*n* = 30), lenticular with a paler equatorial slit in side view. *Sterile cells* (13−) 14–21 (−27) μm × 5–7 (−9) μm elongated, mixed among conidia.

##### Culture characteristics

Ascospores germinated on WA within 24 h. Colonies on PDA reached 8 cm in 7 days at 25 °C, flat, aerial mycelium white. The hyphae in the center are cottony, dense, and there is a thin circle of hyphae at the edge. Reverse grayish white with a dirty white patch.

##### Materials examined

**China**, Guizhou Province, Chishui City, Zhuhai National Forest Park, on dead culms of bamboo, 10 July 2019, Yao Feng, CS19-8 (HKAS 111960, holotype; GZAAS 20–0109, isotype), ex-type living culture CGMCC 3.20134 = GZCC20–0108. *Ibid.*, on dead branch of bamboo, 11 July 2019, Ya-Ya Chen, CS 025 (GZAAS 20–0111), living culture GZCC 20–0109.

##### Notes

Two isolates, representing *Arthrinium septatum*, grouped in a well-supported clade and appear to be distinct from other *Arthrinium* species phylogenetically (Figure 1). *Arthrinium septatum* resembles to *A. biseriale* in having biseriate, broad fusiform to cylindrical ascospores and cylindrical, clavate asci. However, *Arthrinium septatum* differs from *A. biseriale* by having smaller stromata (160–185 μm diam vs. 138–207 μm diam) and asci (75–104 × 17–26 μm vs. 84–116 μm × 18–25 μm).

#### *Arthrinium cyclobalanopsidis* Y. Feng and Jian K. Liu, sp. nov. Figure 5

Index Fungorum number: IF558139

**FIGURE 5:**
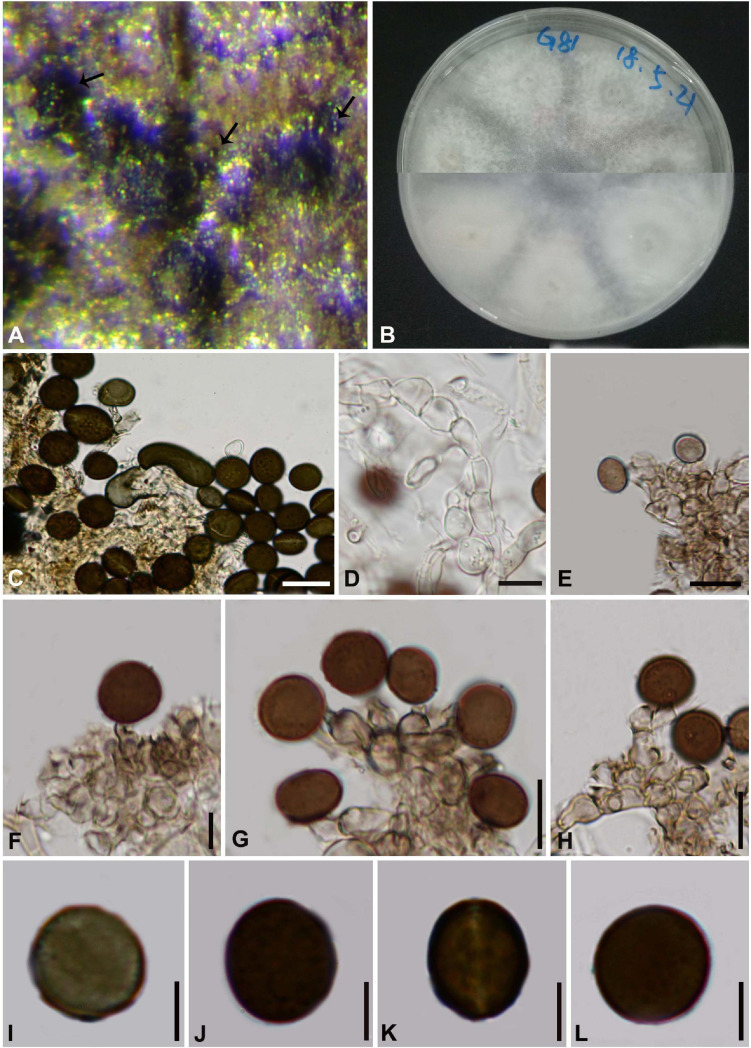
***Arthrinium cyclobalanopsidis* (**HKAS 111963, **holotype) (A)** Conidia masses on host. **(B)** Culture. **(C)** Sterile cell mixed among conidia. **(D)** Hyphae of chain structure. **(E–H)** Conidiogenous cells and conidia. **(I–L)** Conidia. **Scale bars: (C–H)** = 10 μm. **(I–L)** = 5 μm.

Facesoffungi number: FoF 09572

Etymology: The epithet “cyclobalanopsidis” refers to the host plant, *Cyclobalanopsidis glauca* (Thunb.) Oerst.

Holotype: HKAS 111963

*Saprobic* on *Cyclobalanopsidis glauca* (Thunb.). **Sexual morph:** Undetermined. **Asexual morph:** On PDA, *Hyphae* 2.5–5.5 μm in diameter, hyaline, septate, branched with chain structure. *Conidiophores* reduced to the conidiogenous cells. *Conidiogenous cells* 6.0–19.0 μm × 2.5–7.0 μm ($\bar{x}$ = 11.0 μm × 4.5 μm, *n* = 20), aggregated in clusters on hypha, pale brown, ampuliform or cylindrical. *Conidia* 8–12 μm long (*n* = 30), brown, smooth, globose to ellipsoid in surface view, and 10–14 μm long (*n* = 30), lenticular, with a paler equatorial slit in side view. *Sterile cells* elongated, rolled up, sometimes mixed among conidia.

##### Culture characteristics

Conidia germinated on WA within 12 h. Sporulated on PDA, Colonies flat, margin circular, fluffy, sparse, white, with dirty white patches in center, reverse white, with sparse aerial mycelium, reached 8 cm in 7 days at 25°C.

##### Material examined

**China**, Guizhou Province, Qianxinan Buyi and Miao Autonomous Prefecture, Ceheng County, on Leaf of *cyclobalanopsidis glauca* (Thunb.) Oerst., 13 May 2018, Yao Feng, G81 (HKAS 111963, holotype; GZAAS 20–0096, isotype), ex-type living cultures, CGMCC 3.20136 = GZCC 20–0102. *Ibid*., 20 Oct. 2019, Yao Feng, G82 (GZAAS 20–0097), living culture GZCC 20–0103.

##### Notes

Two isolates, representing *Arthrinium cyclobalanopsidis*, cluster together with *A. camelliae-sinensis* which was introduced by Wang et al. (2018) from *Camellia sinensis* (Figure 1). *Arthrinium cyclobalanopsidis* can be distinguished from *A. camelliae-sinensis* (567/572 in ITS; 390/414 in TEF; 715/748 in TUB2). Morphologically, *Arthrinium cyclobalanopsidis* resembles to *A. camelliae-sinensis* in having similar conidia (8–12 μm × 10–14 μm vs. 9.0–13.5 μm × 7.0–12.0 μm), but can be distinguished by its relatively longer conidiogenous cells (6.0–19.0 μm vs. 4.0–9.5 μm).

#### *Arthrinium garethjonesii* D.Q. Dai and H.B. Jiang, Mycosphere 7 (9): 1337 (2017). Figure 6

Saprobic on dead bamboo branch**. Sexual morph**: See Dai et al. (2016). **Asexual morph:** *Sporodochia* on host with hair-like setae, also grow in the gaps of the perithecia and scatter on the surface of the perithecia, black. *Conidiophores* reduced to conidiogenous cells. *Conidiogenous cells* (5−) 6–19 (−20) μm × (2−) 3–5 (−7) μm ($\bar{x}$ = 11 μm × 4 μm, *n* = 20), aggregated in black sporodochia, hyaline to pale brown, smooth, ampulliform. *Conidia* (14–) 16–19 (–20) μm diam, brown, smooth, granular, globose to subglobose in surface view, and (16−) 17–22 (−23) μm diam, with pale equatorial slit in side view.

**FIGURE 6:**
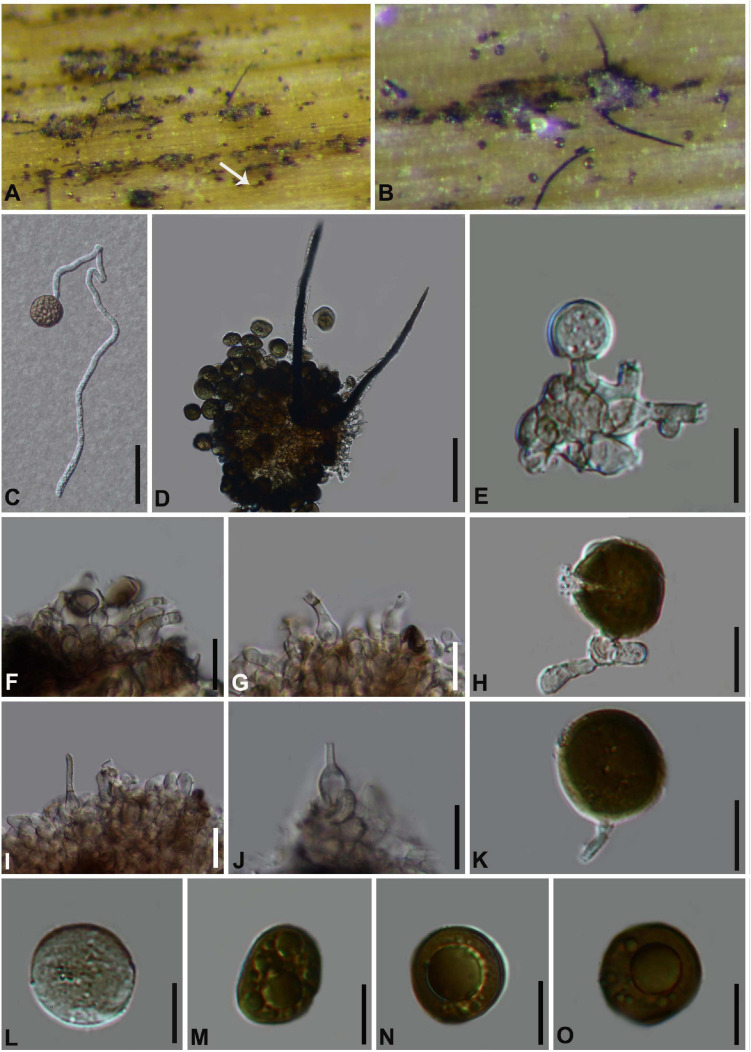
***Arthrinium garethjonesii*** (GZAAS 20–0117) **(A,B)** Appearance of sporodochia on bamboo host. **(C)** Germinating conidia. **(D)** Conidia with setae. **(E–K)** Conidiogenous cells and conidia. **(L–O)** Conidia. **Scale bars: (C)** = 30 μm. **(D)** = 50 μm. **(E)** = 20 μm. **(F,G)** = 10 μm. **(H)** = 5 μm. **(I,J)** = 10 μm. **(K–O)** = 5 μm.

##### Culture characteristics

Conidia germinated on WA within 12 h, colonies fast growing on PDA, reached 8 cm in 7days at 25°C, fluffy, circular, dense, raised at center, white, reverse reddish. Hyphae 2–4 μm diam, hyaline to pale brown, branched, septate.

##### Material examined

**China**, Guizhou Province, Chishui City, Zhuhai National Forest Park, on dead branches of bamboo, 10 July 2019, Yao Feng, CS19-9 (GZAAS 20–0117); living culture GZCC 20–0115.

##### Notes

*Arthrinium garethjonesii* was originally described by Dai et al. (2016) based on the sexual morph from dead bamboo culms (HKAS 96289) collected from Yunnan Province, China. Our phylogenetic result (Figure 1) indicates that our collection is identical to *Arthrinium garethjonesii* and we report its asexual morph for the first time in this study.

#### *Arthrinium guizhouense* M. Wang and L. Cai, Mycokeys 34: 13 (2018). Figure 7

*Saprobic* on dead bamboo culms, forming black, lenticular spots on the host surface, stromata breaking through raised cracks with black center. **Sexual morph:** *Stromata* solitary to gregarious, immersed to erumpent, fusiform, with long axis broken at the top by one cracks. *Ascomata* 188–220 μm high × 170–200 μm wide, uniseriate or irregularly arranged beneath stromata, pseudothecial, black, globose to subglobose. *Peridium* composed of 5 or 6 layers of brown cells arranged in *textura angularis*, *Hamathecium paraphyses* 3–5 μm, hyaline, hyphae-like, septate. *Asci* (80−) 94–106 (−107) μm × (20−) 21–23 (−24) μm, 8-spored, clavate, apically rounded, broadly cylindrical, with an indistinct pedicel. *Ascospores* (24−) 25–32 (−33) μm × (6−) 7–9 (−10) μm ($\bar{x}$ = 31 μm × 8 μm, *n* = 30), apiosporic, clavate to fusiform with narrowly rounded ends, composed of a large upper cell and small lower cell, hyaline, smooth-walled, surrounded an inconspicuous gelatinous sheath. **Asexual morph:** *Hyphae* 2.5–7.5 μm diam, hyaline, branched, septate. *Conidiophores* reduced to conidiogenous cells. *Conidiogenous cells* 4–12 μm × 2–5 μm ($\bar{x}$ = 8 μm × 3 μm, *n* = 20), erect, aggregated in clusters on hyphae, pale brown, smooth, subglobose, ampulliform or doliiform. *Conidia* 5–8 μm long (*n* = 30), dark brown to black, smooth, globose or subglobose, and 6–8 μm long (*n* = 30), lenticular, with a paler equatorial slit in side view. *Sterile cells* elongated, rolled up, sometimes mixed among conidia.

**FIGURE 7:**
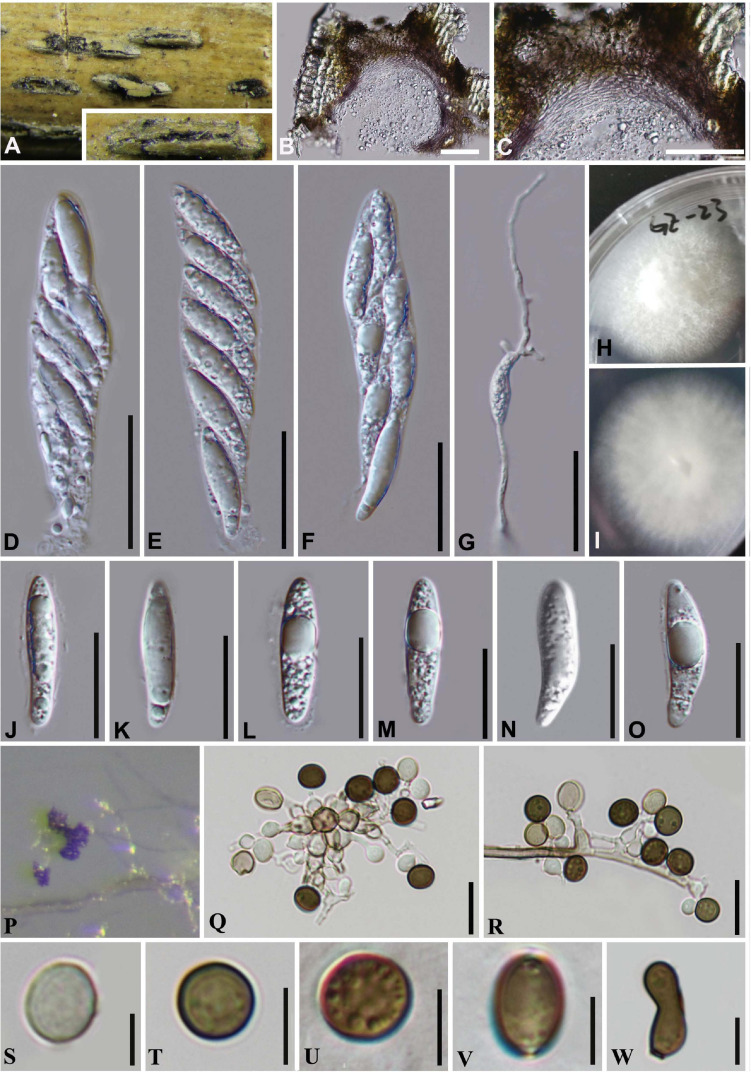
***Arthrinium guizhouense*** (GZAAS 20–0114). **(A)** Appearance of stromata on bamboo host. **(B)** Vertical section of stroma. **(C)** Peridium. **(D–F)** Asci. **(G)** Germinating ascospore. **(H,I)** Culture. **(J–O)** Ascospore. **(P)** Colony on WA producing conidia masses. **(Q,R)** Conidiogenous cells giving rise to conidia. **(S–V)** Conidia. **(W)** Sterile cell. **Scale bars: (B,C)** = 50 μm. **(D–F)** = 30 μm. **(G)** = 50 μm. **(J–O)** = 20 μm. **(Q,R) =** 10 μm. **(S–W) =** 5 μm.

##### Culture characteristics

On PDA, colonies very fast, reached 8 cm in 8 days at 25°C, velvety, circular, with regular edge, middle densely and raised white, dense at above the from margin, aerial mycelia, surface initially white, became grayish and reverse white.

##### Material examined

**China**, Guangdong Province, Guangzhou City, on decaying bamboo culms, 03 Sep. 2019, Yao Feng, GZ 23 (GZAAS 20–0114); living culture GZCC 20–0114.

##### Notes

*Arthrinium guizhouense* was introduced from air in a karst cave (asexual morph was provided from the culture) in Guizhou province, China by Wang et al. (2018). In this study, one collection was found as saprobe on bamboo in Guangzhou, China and it is identified as *A. guizhouense* based on the phylogeny and morphology evidences. In addition, our new collection provides the sexual morph which only the asexual morph was illustrated by Wang et al. (2018) and Senanayake et al. (2020a).

#### *Arthrinium phyllostachium* C.L. Yang, X.L. Xu and K.D. Hyde, Phytotaxa 406 (2): 102 (2019). Figure 8

*Saprobic* on dead bamboo culms, **Sexual morph:** *Stromata* scattered to gregarious, immersed to erumpent, later becoming superficial, dark brown to black, fusiform, forming a slit-like opening at the apex, with stromata breaking through raised cracks with black center. *Ascomata* 135–185 μm high, 157–215 μm diam, multi-loculate, with a periphysate ostiole, arranged in rows, clustered, gregarious, ampulliform, dark brown to black. *Peridium* 20–25 μm wide, composed of several layers of dark brown to brown cells of *textura angularis*. *Hamathecium* 3–5 μm wide, comprising dense, hyaline, septate paraphyses, hyphae-like. *Asci* 66–98 × 17–27 ($\bar{x}$ = 85 μm × 21 μm, *n* = 20), 8-spored, unitunicate, clavate, apedicellate, apically rounded. *Ascospores* 29–34 μm × 7–10 μm ($\bar{x}$ = 32 μm × 9 μm, *n* = 30), biseriate, elliptical, hyaline, 1-septate, constricted at the septum, mostly curved at the lower cell, rarely straight, with a large upper cell and a small lower cell, smooth-walled, with a shallow gelatinous sheath. **Asexual morph:** On WA, *Hyphae* 1.5–4.0 μm in diameter, hyaline, septate, branched. *Conidiophores* reduced to the conidiogenous cells. *Conidiogenous cells* (8−) 9–28 (−31.5) μm × (1.5−) 2–4 (−6) μm ($\bar{x}$ = 20 μm × 3 μm, *n* = 20), aggregated in clusters on hypha or solitary, erect, ampuliform or cylindrical, arising holoblastically from vegetative hyphae, monoblastic, or polyblastic, sympodial, terminal, cylindrical to clavate, ampulliform, hyaline, smooth, thin-walled. *Conidia* 5–8 μm long (*n* = 30), brown, smooth, globose to ellipsoid in surface view, and 7–9 μm long (*n* = 30), lenticular, with a paler equatorial slit in side view. *Sterile cells* light brown, elongated, and occasionally irregularly angled.

**FIGURE 8:**
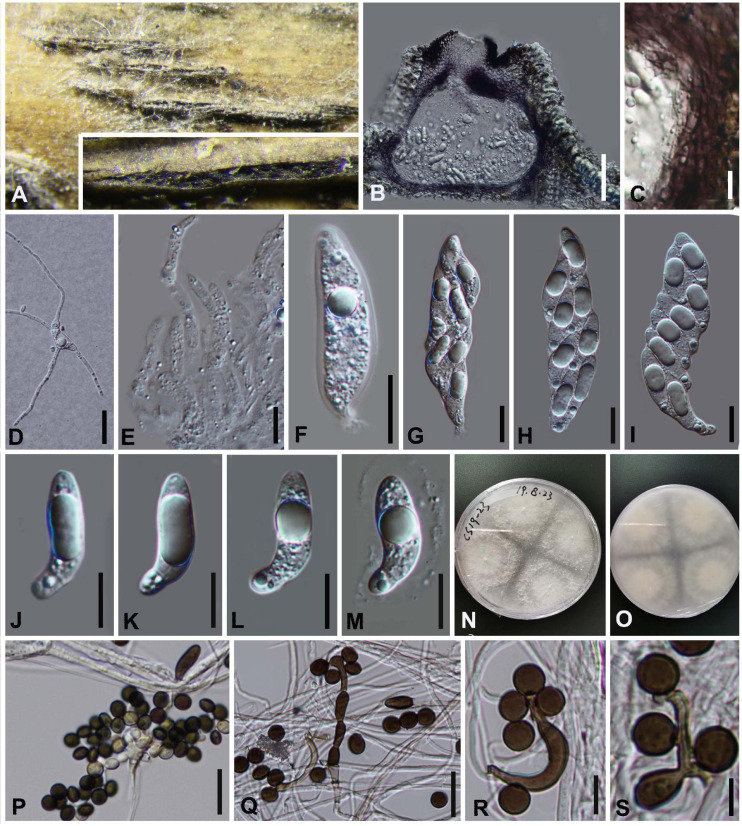
***Arthrinium phyllostachium*** (GZAAS 20–0112) **(A)** Appearance of stromata on bamboo host. **(B)** Vertical section of stroma. **(C)** Peridium. **(D)** Germinating ascospore. **(E)** Paraphyses. **(F–I)** Asci. (**J–M)** Ascospore. (**N**,**O)** Culture. **(P–S)** Conidiogenous cells and conidia. **Scale bars: (B)** = 50 μm. **(C)** = 10 μm. **(D)** = 20 μm. **(E)** = 20 μm. **(F–I)** = 30 μm. **(J–M)** = 10 μm. **(P,Q)** = 20 μm. **(R–S)** = 10 μm.

##### Culture characters

Ascospores germinated on WA within 24 h and germ tubes produced from Upper, middle and lower end. Colonies fast growed on PDA at 25°C, flat, spreading, the middle is white, smooth, the edge surface tapetum, gray-white, indistinct aerial hyphae, the reverse side is yellowish in the middle, and the edge is grayish white.

##### Materials examined

**China**, Guizhou Province, Chishui City, Zhuhai National Forest Park, on dead culms of bamboo, 10 July 2019, Yao Feng, CS 19-23 (GZAAS 20–0112), living culture GZCC 20–0111; *Ibid*., Chishui National Scenic Area, on dead culms of bamboo, 10 July 2019, Ya-Ya Chen, CS004 (GZAAS 20–0113), living culture GZCC 20–0112.

##### Notes

*Arthrinium phyllostachium* was introduced by Yang et al. (2019) based on the asexual morph and phylogeny analyses. It was collected from culms of *Phyllostachys heteroclada* (Poaceae) in China (Yang et al., 2019). The phylogenetic results showed that our new collections are identical to *A*. *phyllostachium*, Yang et al. (2019) only provided the asexual morph and the sexual morph is given in this study.

#### *Arthrinium pseudoparenchymaticum* M. Wang and L. Cai, Mycokeys 34 (1): 17 (2018), Figure 9

*Saprobic* on dead bamboo culms, forming black, lenticular spots on the host surface, with stromata breaking through raised cracks with black center. **Sexual morph:** *Ascomata* 187–242 μm high × 242–373 μm wide, uniseriate, or irregularly arranged beneath stromata, black, globose to subglobose with a flattened base. *Peridium* composed of 5–7 layers of brown cells arranged in *textura angularis*, with a conspicuous perfusate ostiole. *Hamathecium paraphyses* 4–8 μm, hyphae-like, septa hyaline. *Asci* (95−) 107–110 (−133) μm × 23–25 (−27) μm, 8-spored, broadly cylindrical, clavate or subglobose, apically rounded, with an indistinct pedicel. *Ascospores* (35–) 35–43 (–44) × (10–) 11–13 μm ($\bar{x}$ = 41 × 12, *n* = 30), apiosporic, clavate to fusiform with narrowly rounded ends, composed of a large upper cell and small lower cell, hyaline, smooth-walled, surrounded by a gelatinous sheath. **Asexual morph:** On WA, *Hyphae* 1.5–4 μm diam, hyaline to pale brown, branched, septate. *Conidiophore* extends from the vegetative hypha, up to 60 μm long. *Conidiogenous cells* 10.0–40.0 μm × 3.0–6.0 μm, scattered in clusters on hyphae, smooth, unbranched, hyaline to pale yellow, smooth, erect, subcylindrical. *Conidia* 17–27 μm × 17–21 μm ($\bar{x}$ = 23 μm × 19 μm, *n* = 30), pale to light brown, smooth, globose to subglobose, sometimes lobed or dentate, polygonal or irregular in surface view.

**FIGURE 9:**
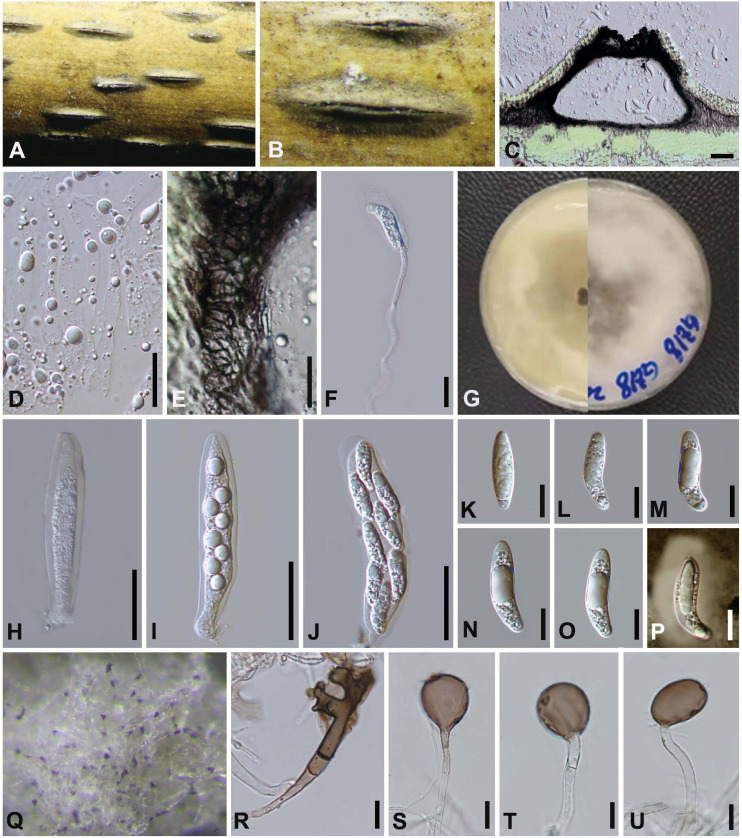
***Arthrinium pseudoparenchymaticum*** (GZAAS 20–0115) **(A,B)** Appearance of stromata on bamboo host. **(C)** Vertical section of stroma. **(D)** Paraphyses. **(E)** Peridium. **(F)** Germinating ascospore. **(G)** Culture. **(H–J)** Asci. **(K–O)** Ascospore. **(P)** Ascospore in Indian ink and present clear gelatinous sheath. **(Q)** Colony on WA producing conidia masses. **(R)** Dentate conidia. **(S–U)** Conidiogenous cells giving rise to conidia. **Scale bars: (C)** = 50 μm. **(D–F)** = 20 μm. **(H–J)** = 30 μm. **(K–P)** = 15 μm. **(R–U)** = 15 μm.

##### Culture characteristics

Ascospores germinating on WA within 24 h and germ tubes produced from upper. Colonies fast growing on PDA at 25°C, under 12 h light/12 h dark, cottony, circular, sparse, raised, with irregular edge, white in center.

##### Material examined

**China**, Guangdong Province, Guangzhou City, on decaying bamboo culms, 3 Sep. 2019, Yao Feng, GZ18 (GZAAS 20–0115), living culture GZCC20–0117.

##### Notes

*Arthrinium pseudoparenchymaticum* was introduced by Wang et al. (2018) based on the asexual morph characters and phylogeny analyses. It was originally collected from bamboo in China (Wang et al., 2018). In this study, a fresh specimen was collected and it is identical to *A. pseudoparenchymaticum* (Figure 1), both sexual and asexual morphs were described and illustrated (Figure 9).

#### *Arthrinium arundinis* (Corda) Dyko and B. Sutton, Mycotaxon 8: 119 (1979). Figure 10.

*Saprobic* on dead bamboo culms. **Asexual morph:** On PDA, *Hyphae* 2–3 μm diam, consisting of smooth, hyaline, branched, septate. *Conidiophores* reduced to conidiogenous cells. *Conidiogenous cells* 3–8 μm × 2–5 μm, aggregated in clusters on hyphae, pale brown, smooth, ampulliform. *Conidia* (4–) 5–6 (–7) μm, brown, smooth, globose in surface view, and (4−) 5–7 (−8) μm diam, lenticular with pale equatorial slit in side view. *Sterile cells* at times intermingled among conidia. **Sexual morph:** Undetermined.

**FIGURE 10:**
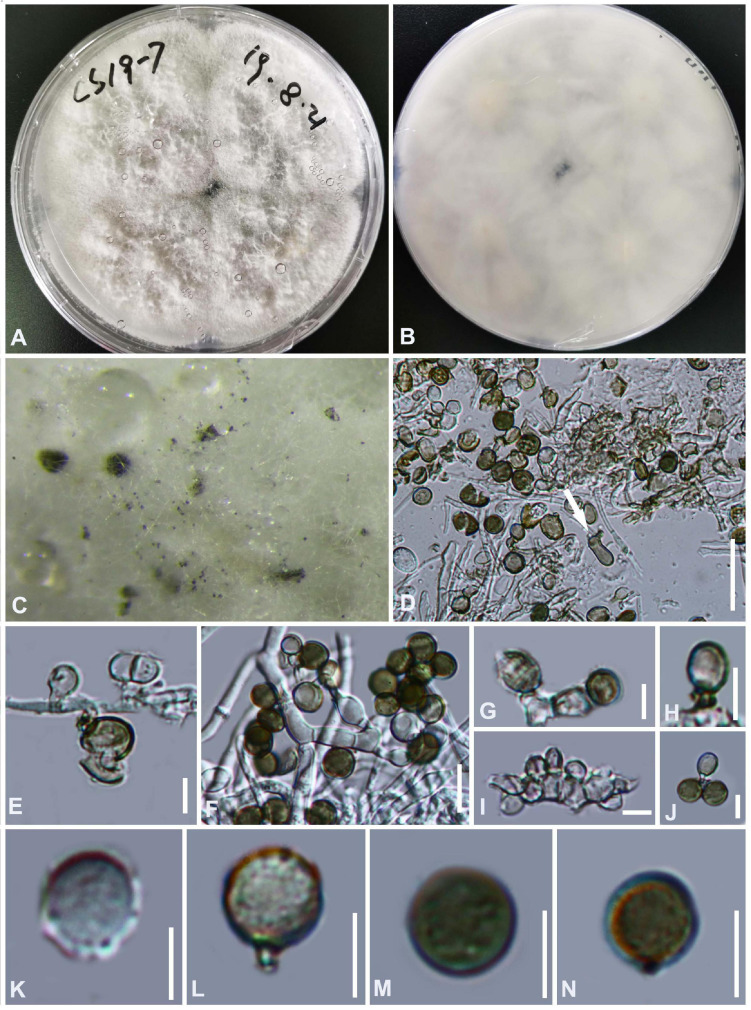
***Arthrinium arundinis*** (GZAAS 20–0116) **(A,B)** culture. **(C)** Colonies on culture. **(D)** Sterile cell mixed among conidia. **(E–J)** Conidiogenous cells and conidia. **(K–N)** Conidia. **Scale bars: (D)** = 50 μm. **(E)** = 5 μm. **(F)** = 10 μm. **(G–N)** = 5 μm.

##### Culture characteristics

The colony is flat, cotton-like, thick and dense, with sparse aerial mycelia. The surface of PDA is white and the reverse side is grayish white.

##### Materials examined

**China**, Guizhou Province, Chishui City, Zhuhai National Forest Park, on dead culms of bamboo, 10 July 2019, Yao Feng, CS 19-7 (GZAAS 20–0116), living culture GZCC 20–0116.

##### Notes

Our collection clusters together with the isolates of *Arthrinium arundinis* (Figure 1) and its morphology lines up with the type species. Therefore we identify it as *Arthrinium arundinis*.

#### *Arthrinium hydei* Crous, IMA Fungus 4 (1): 142 (2013). Figure 11

*Saprobic* on bamboo leaves. **Asexual morph:** *Colonies* on the host punctiform, pulvinate, blackish brown. *Conidiophores* pale brown, smooth, transversely septate, subcylindrical. *Conidiogenous cells* 5–15 × 3–6 μm, brown, smooth, subcylindrical to doliiform to lageniform. *Conidia* (13−) 14–19 (−20) μm diam in surface view, brown, roughened, globose, and 15–20 μm diam, lenticular with pale equatorial slit in side view. **Sexual morph:** Undetermined.

**FIGURE 11:**
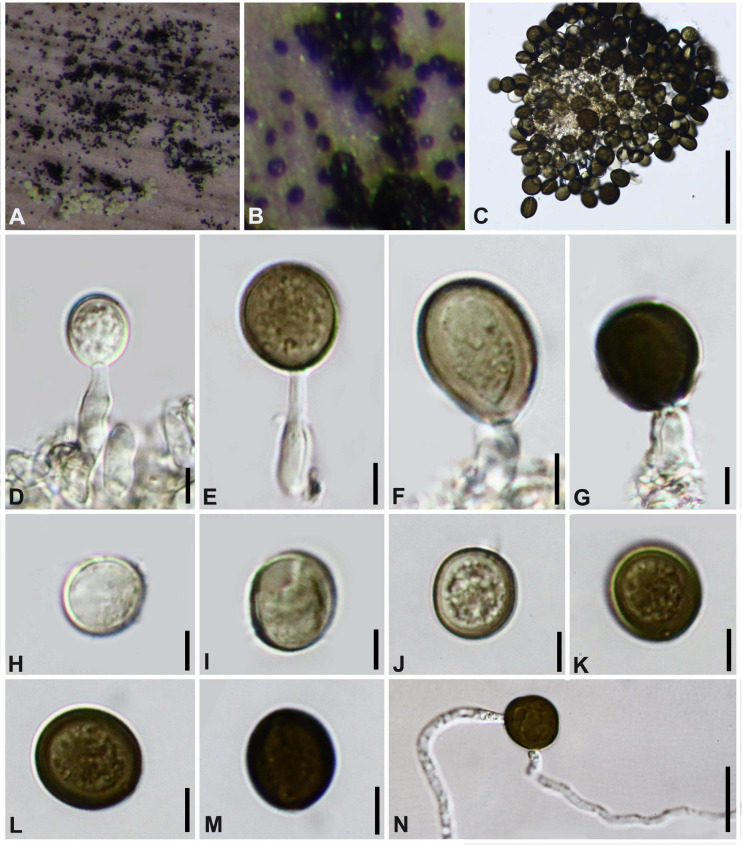
***Arthrinium hydei*** (GZAAS 20–0098) **(A**,**B)** Colonies on substrate. **(C–F)** Conidiogenous cells and conidia. **(G–M)** Conidia. **(N)** Germinating conidia. **Scale bars: (C)** = 50 μm. **(D–M)** = 5 μm. **(N)** = 25 μm.

##### Culture characteristics

Colonies flat, spreading, with sparse aerial mycelium. On PDA surface and reverse pale luteous. Mycelium consisting of smooth, hyaline to pale brown, branched, septate, 2.0–4.5 μm diam hyphae.

##### Materials examined

**China**, Guizhou Province, Guiyang City, Baihua Lake, on bamboo leaves, 20 April 2018, Yao Feng, 67 (GZAAS 20–0098), living culture GZCC20–0113.

##### Notes

This collection is identified as *Arthrinium hydei* based on both morphological characters and molecular data. Crous and Groenewald (2013) originally described *A. hydei* based on the asexual morph from a culture (CBS 114990) which was isolated from bamboo culms in Hong Kong, China; we found this species in Guizhou from the substrate in nature with its asexual morph.

#### *Arthrinium neosubglobosa* D.Q. Dai and H.B. Jiang, Mycosphere 7 (9): 1337 (2017) Figure 12.

*Saprobic* on dead bamboo culms. **Sexual morph:** *Stromata* scattered to gregarious, superficial to raised, with a slit-like opening, dark brown to black, naviculate, with black papillate ostiole, multi-loculate. *Ascomata* 205–328 μm high, 168–345 μm, perithecial, arranged in a row, immersed in stromata, later becoming erumpent through host surface to superficial, obpyriform to ampulliform, dark brown, membranous. Ostiole raised from center of *Ascomata*, internally lined with periphyses. *Peridium* 4 layers, outer layer composed of dark brown, cells of *textura prismatica*, inner layer thin, with hyaline cells of *textura angularis*. *Hamathecium* 4.0–5.5 μm wide, comprising dense paraphyses, indistinctly aseptate, unbranched, not anastomosing, filamentous, clustered embedded in gelatinous matrix. *Asci* 80–119 μm × 20–37 μm ($\bar{x}$ = 97 μm × 28 μm, *n* = 20), 8-spored, unitunicate, clavate, with a short pedicel, apically rounded. *Ascospores* 25–36 μm × 11–15 μm ($\bar{x}$ = 29 μm × 13 μm, *n* = 30), 2-seriate, elliptical, hyaline, 1-septate, constricted at the septum, mostly curved at the lower cell, rarely straight, with a large upper cell and a small lower cell, smoothwalled, 1-guttulate, with a shallow 8–12 μm thick gelatinous sheath. **Asexual morph:** Undetermined.

**FIGURE 12:**
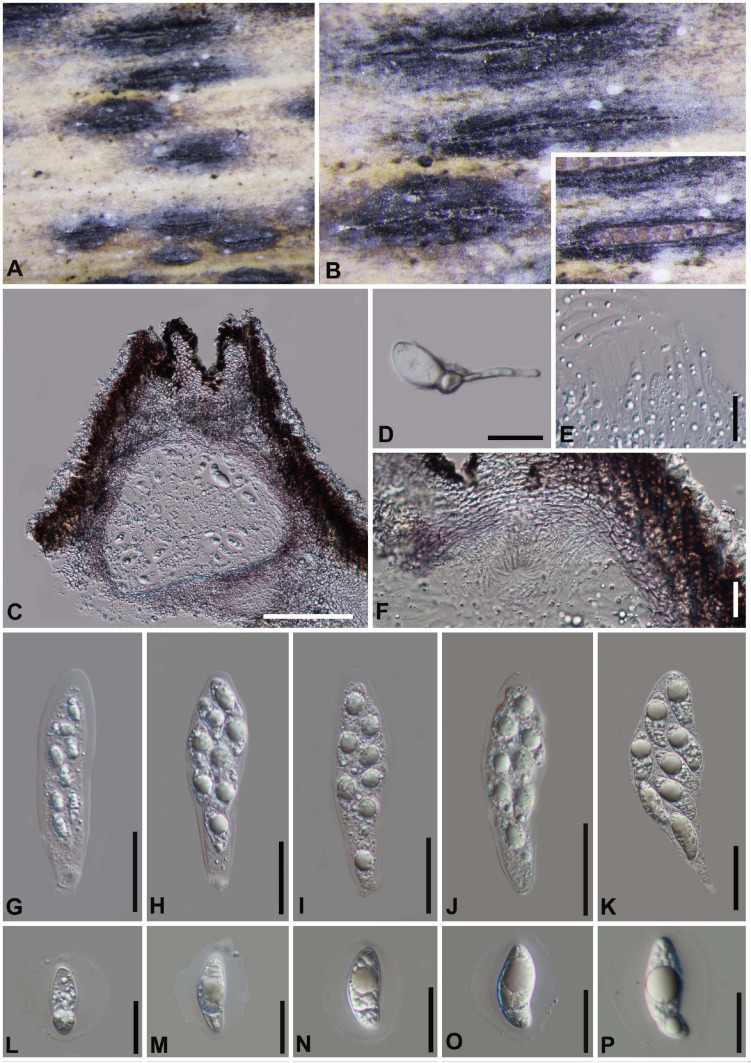
***Arthrinium neosubglobosa*** (GZAAS 20–0099). **(A,B)** Appearance of stromata on bamboo host. **(C)** Vertical section of stroma. **(D)** Germinating ascospore. **(E)** Paraphyses. **(F)** Peridium. **(G–K)** Asci. **(L–P)** Ascospore. **Scale bars: (C)** = 100 μm. **(D–F)** = 20 μm. **(G–K)** = 30 μm. **(L–P)** = 20 μm.

##### Materials examined

**China**, Guizhou Province, Zunyi City, Daozhen County, on dead culms of bamboo, 15 August 2018, Yao Feng, DZ22 (GZAAS 20–0099).

##### Notes

*Arthrinium neosubglobosa* was introduced by Dai et al. (2016) based on the sexual morph characters and phylogeny analyses. The pure culture was attempted by single spore isolation and the DNA was extracted directly from the fruiting body, the new collection is identified as *A*. *neosubglobosa* based on the phylogeny (Figure 1) and morphology evidences.

## Discussion

*Arthrinium* species have been reported from many hosts, includes hive-stored pollen lichens, marine algae, soil debris, gut of insects and nodules of human skin (Sharma et al., 2014; Crous et al., 2015, Senanayake et al., 2015; Wijayawardene et al., 2017, Zhao et al., 2018), it can be concluded that *Arthrinium* is ecologically diverse. Bamboo, as one of the most reported host, is a gramineous plant integrating economy and ornamental value (Gratani et al., 2008; Kelchner and Bamboo Phylogeny Group, 2013; Dai et al., 2016, 2017; Jiang et al., 2018, 2019, 2020; Wang et al., 2018; Yang et al., 2019), there is more than 115 genera with approximately 1,450 species. According to incomplete statistics, more than 1,100 species of fungi on bamboo were reported (Hyde et al., 2002a, b; Dai et al., 2016, 2017; Senanayake et al., 2020a; Tang et al., 2020; Wijesinghe et al., 2020). It is of great significance to excavate and identify the fungi on bamboo.

Several studies have shown (Crous and Groenewald, 2013; Dai et al., 2016, Dai et al., 2017; Wang et al., 2018; Yang et al., 2019) that it is difficult to identify the *Arthrinium* species solely rely on morphology and the multi-gene phylogenetic analyses are needed in the identification and classification of *Arthrinium*. The morphology of conidia is variable which can be depending on the period of incubation on different habitats, for example, *A. biseriale*, *A. gelatinosum* and *A. septatum*, are very similar in morphology, but molecular data distinguish them into different species; Our collection *A. pseudoparenchymaticum* differs from the type specimen (LC 8173) in the morphology of conidiophores, the size of conidiogenous cells is also different, while the molecular data supported them as the same species. These results are in agreement with the previous observations and publications (Crous and Groenewald, 2013; Dai et al., 2016, Dai et al., 2017; Wang et al., 2018, Yang et al., 2019). In addition, as a high diverse group, it is also difficult to distinguish species within *Arthrinium* by only using ITS and LSU gene regions, and the protein genes (TEF and TUB2) are not available for many species in the genus which bring potential problem once the new or existing taxa are introduced and identified, respectively. For example the absent of the protein genes (TEF and TUB2) of *Arthrinium garethjonesii* would bring the troubles in identification of *Arthrinium setostromum* as it is hard to confirm whether they are same species or not as they are identical in ITS and LSU gene regions, as well as the close phylogenetic relationship (Figure 1). It would be necessary to provide protein genes when new taxa are introduced in these well-study and diverse groups.

## Data Availability Statement

The data presented in the study can be found in the Genbank. The accession numbers of the sequences deposited in GenBank are ITS: MW481705—MW481721; LSU: MW478885–MW478901; TEF: MW522938–MW522954; and TUB: MW522955–MW522969.

## Author Contributions

YF and J-KL: conceptualization. YF and Y-YC: methodology. YF, C-GL, and J-KL: formal analysis. YF, Y-YC, M-MX, and J-KL: resources. YF: writing—original draft preparation. C-GL, Z-YL, and J-KL writing—review and editing. Z-YL and J-KL: supervision. All authors approved to publish the version of final manuscript.

## Conflict of Interest

The authors declare that the research was conducted in the absence of any commercial or financial relationships that could be construed as a potential conflict of interest.

## Footnotes

1 http://www.facesoffungi.org/

2 https://blast.ncbi.nlm.nih.gov/Blast.cgi
